# A Projector-Based Augmented Reality Navigation System for Computer-Assisted Surgery

**DOI:** 10.3390/s21092931

**Published:** 2021-04-22

**Authors:** Yuan Gao, Yuyun Zhao, Le Xie, Guoyan Zheng

**Affiliations:** 1 Institute of Forming Technology & Equipment, Shanghai Jiao Tong University, Shanghai 200030, China; gao.yuan@sjtu.edu.cn; 2 Institute of Medical Robotics, Shanghai Jiao Tong University, Shanghai 200240, China; yuyun.zhao@sjtu.edu.cn; 3 School of Biomedical Engineering, Shanghai Jiao Tong University, Shanghai 200240, China

**Keywords:** spatial augmented reality, image overlay projection, computer-assisted surgery, surgical navigation

## Abstract

In the medical field, guidance to follow the surgical plan is crucial. Image overlay projection is a solution to link the surgical plan with the patient. It realizes augmented reality (AR) by projecting computer-generated image on the surface of the target through a projector, which can visualize additional information to the scene. By overlaying anatomical information or surgical plans on the surgery area, projection helps to enhance the surgeon’s understanding of the anatomical structure, and intuitively visualizes the surgical target and key structures of the operation, and avoid the surgeon’s sight diversion between monitor and patient. However, it still remains a challenge to project the surgical navigation information on the target precisely and efficiently. In this study, we propose a projector-based surgical navigation system. Through the gray code-based calibration method, the projector can be calibrated with a camera and then be integrated with an optical spatial locator, so that the navigation information of the operation can be accurately projected onto the target area. We validated the projection accuracy of the system through back projection, with average projection error of 3.37 pixels in *x* direction and 1.51 pixels in *y* direction, and model projection with an average position error of 1.03 ± 0.43 mm, and carried out puncture experiments using the system with correct rate of 99%, and qualitatively analyzed the system’s performance through the questionnaire. The results demonstrate the efficacy of our proposed AR system.

## 1. Introduction

The development of medical imaging technology, including magnetic resonance (MR), computed tomography (CT), ultrasonic tomography, etc., can help to provide patients’ imaging data, so as to locate the lesions and design the surgical plan [1]. How the digital surgical plan can be linked to real patients intraoperatively is very important. At early stages, surgeons completely relied on their cognitive experience of the patient’s anatomy to perform operations, which is inefficient and unsafe. Later, surgeons used frame-type three-dimensional mechanisms to guide doctors to perform operations, which requires placing a heavy mechanical device on the patient [2]. With the development of spatial positioning technology and image registration technology, image-guided surgery has gradually become the mainstream, which is a discipline where computer technology is applied preoperatively, intraoperatively and postoperatively to improve the outcome of surgical procedures [3,4]. It allows the surgeon to measure the intraoperative placement of the medical components in real time, making sure that the operation procedure adheres to the preoperative plan [5]. The image guidance method currently used is mainly to display the surgical plan through the traditional flat-panel display. However, when the display and the patient are separated, it means that the surgical plan is not visually unified with the patient. The surgeon needs to constantly switch eyesight between the patient and the screen, which will easily lead to distraction and a decline in the understanding of the surgical plan. The emergence of AR has become a good way to solve the problem.

Since Boeing researcher Tom Caudell first proposed the term of AR in 1990 [6], the concept of AR has been gradually accepted. In 1997, Azuma et al. defined the AR with three technical points [7]: (1) Combination of real and virtual views; (2) Real-time interactions; (3) Views registered in 3D. AR is regarded as an advanced interface that combines supplemental information with the real-world environment and has made a great breakthrough over the last few decades, with the rapid development of computer and image processing technologies. AR has been used in the entertainment [8], education [9], military [10], manufacturing [11] and medical fields (training [12], as well as preoperative planning [13], and intraoperative guidance [14]). AR-based surgical navigation systems help to improve surgeons’ performance during surgical procedures by overlaying image data and surgical plan on the patient. There are various approaches to display virtual content over the real environment in AR, which could be mainly divided into video see-through, optical see-through and spatial augmented reality [15].

Video see-through AR merges live record video streams with computer-generated graphics and then displays the mixed result on the screen. This approach is the easiest and most widely used, however, the covered field of view (FOV) is limited by the size of screen and camera. The performance of this approach is sensitive to the latency and the resolution of the video, and is also affected by lens distortion. Furthermore, if the AR system is broken, the user may not be able to see the real environment, which could cause danger in the surgery. In 2005, Mischkowski et al. used a tracked portable screen with a digital camera on its backside to implement the video-through AR in cranio-maxillofacial surgery [16], and the surgeon holds the screen to carry out the surgery, yet it is still difficult for the surgeon to conduct the surgery only seeing the video, with no real environment.

Optical see-through AR generates computer graphics appearing on a semi-reflective mirror or transparent liquid crystal display (LCD) screen within the real environment. Surgeons can always see the real environment. The FOV is usually limited by the optical technology. The surgeon’s eyes are forced to continuously shift focus between virtual objects and the real patient at different depths. Due to the optical see-through devices’ calibration related to the human eyes, the calibration is challenging and unstable. In 2014, Wang et al. applied augmented reality in the oral and maxillofacial surgery, in which they used a half-silvered mirror as the 3D display media [17]. The surgeon’s view is restricted to the size of the mirror, which blocks some details during surgery due to the light absorption of the mirror, and the proposed device takes up too much space in the operation room. In 2018, Condino et al. proposed a head-mounted display (HMD)-based AR system for the orthopedic open surgery [12]. The HMD could visualize hologram at a specific position, but the hologram’s depth is hard to recognized and the hologram must be displayed at a distance away from the user due to the optical limitation of HMD, and the HMD puts an extra burden onto the surgeon.

Spatial augmented reality (SAR) integrates the generated virtual view into the real environment by projecting images directly on physical objects’ surface using a projector. Restrictions of the display are constrained to the size, shape, and color of the physical objects’ surface. The key point of SAR is to project the image onto target accurately. Compared with the other two methods, SAR improves ergonomics, and provides a theoretically unlimited field of view, a scalable resolution, and an easier eye accommodation due to the virtual objects being typically rendered near their real-world location [15]. Furthermore, compared to body-attached AR, the projector-based AR systems bring no burden to the surgeon.

In this paper, considering all the aforementioned comparison, we developed a projector-based AR navigation system for surgical guidance and designed practical application of puncture surgery. In this system, the projector is calibrated with a camera based on gray code projection and then be integrated with an optical tracker. The surgical navigation information is accurately projected onto the target operation area. Finally, we validated the performance of the proposed system.

## 2. Related Work

Some researchers have conducted research on the application of spatial augmented reality in medical treatment. In 2003, Tardif et al. proposed a projector-camera-based AR system which corrects the distortion of the view captured by camera and then projects the corrected picture onto the patient [18], however, no practical application or quantitative experiment was conducted in this study.

In 2007, Krempien et al. designed an AR navigation system using a projector in interstitial brachytherapy [19]; his study provided good ideas about interaction design, but did not mention how to integrate the projector with tracking system, and the projector worked with two cameras but cannot move during operation, due to the fact that once the projector is moved, the registration needs to be done again, which may increase operation time and risk.

Gavaghan et al. proposed a handheld projector to visualize the surgical navigation data in 2011 [20]. This study completed qualitative performance evaluations, and provided inspiration about the applications of the system, but their rendering environment did not consider the deviation between the center of the lens and the center of the image sensor.

Wu et al. applied the SAR onto the real-time advanced spinal surgery in 2014 [21]. By projecting the spine onto the patient’s back, the surgeon can see the entry point. Several round black markers tracked by the camera were pasted on the patient’s skin in advance, which acted as the feature points to overlay the image with the patient. This study did not discuss whether the image can follow the patient in real time when the patient is moving.

There are also some other interesting applications of SAR. Laviole et al. applied SAR to enhance physical artistic creation in 2012 [22]. The SAR projections were combined with an optical see-through near-eye display by Benko et al., in 2015 [23] and Hamasaki et al., in 2018 [24]. Punpongsanon et al. used SAR to visualize the manipulation haptic softness perception in 2015 [25].

The projector’s calibration is the prerequisite to ensure the accurate overlapping of the projection. Gray code was first proposed in 1941 in Bell Labs [26]. Generally, gray code is used in digital communications, position encoders and structured light 3D scanners [27,28,29]. Here, gray code projection is applied in the calibration of the projector.

## 3. Materials and Methods

### 3.1. System Overview

**Figure 1:**
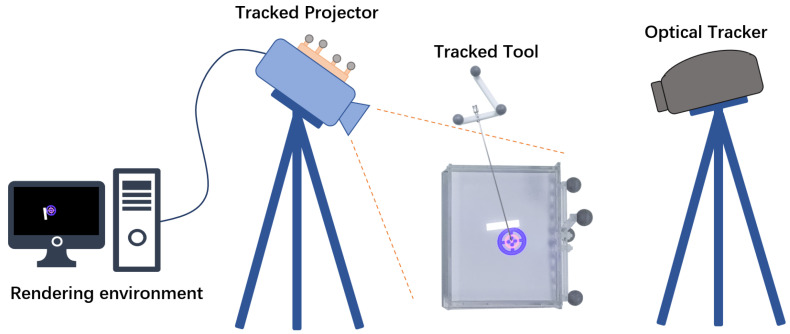
Overview of the system. The surgeon carries out puncture operation under navigation.

The overall setup of our projector-based AR system is shown in Figure 1. The system contains a projector (J6s, JmGo, China), an optical tracking system (OTS) (Polaris Vega, Northern Digital Inc., Waterloo, ON, Canada), and surgical tool. The projector is based on digital light processing (DLP) technology that uses an array of microscopic mirrors reflecting the light. The projector has a resolution of 1920 × 1080 pixels and 1100 ANSI lumens. An optical reference (comprised of four passive sphere markers) was designed and attached to the projector rigidly to be tracked spatially by OTS.

**Figure 2:**
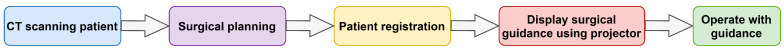
AR guided workflow for the operation.

In the proposed system, the patient’s image data are acquired through CT scanning. The surgeon makes surgical planning based on the image data, and obtains a three-dimensional model based on the reconstruction from image data. The image and surgical plan are imported into a workstation for processing, then are sent to the projector, which will cast navigation information onto the surgery area. The surgeon performs the operation with the guidance of the projected information. The work flow is as shown in Figure 2.

### 3.2. Transformations among System Components

**Figure 3:**
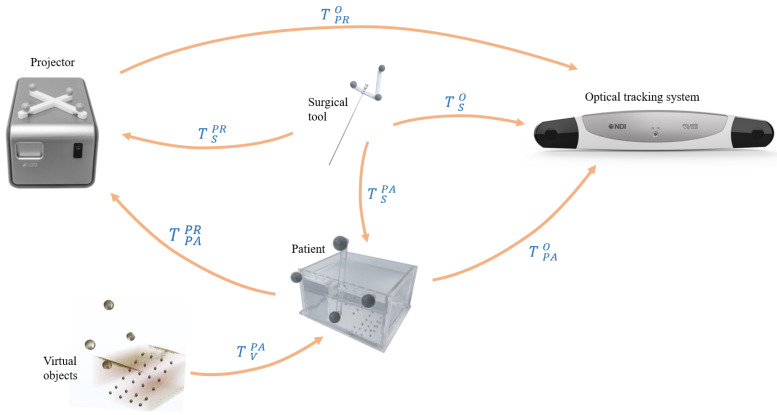
Different coordinate systems involved in our system: the virtual objects were registered to the patient, and the patient was then aligned with the projector’s reference, which is also the base of the virtual environment. The surgical tool was aligned to the projector’s reference for display, and aligned to the patient to compute the deviation.

Different components in the system have their own coordinate systems, which need to be unified to work together. To accurately overlay the projected image on the patient’s surgery area in the right position, the projector needs to be calibrated and the patient needs to be registered. Furthermore, to enable the navigation information to follow the patient’s potential movement, the projector and the patient are tracked in real time. At the same time, in order to connect surgical tool with AR scenes, the surgical tool has to be calibrated and tracked. All the transformations in the system are described as follows, as shown in Figure 3.

- $T_{PR}^{O}$: the transformation from projector to OTS.
- $T_{S}^{O}$: the transformation from surgical tool to OTS.
- $T_{PA}^{O}$: the transformation from patient to OTS.
- $T_{S}^{PA}$: the transformation from surgical tool to patient.
- $T_{V}^{PA}$: the transformation from virtual objects to patient.
- $T_{PA}^{PR}$: the transformation from patient to projector.
- $T_{S}^{PR}$: the transformation from surgical tool to projector.

The projector, the patient and surgical tool are all fixed with markers, which are tracked by the OTS in real time. The local coordinate established on the tracked marker rigidly attached to the projector is set as the world coordinate system. Through the registration of the patient, the transformation from image to patient $T_{V}^{PA}$ is obtained. To correctly project the patient image through the projector, the image must be converted to the projector’s local coordinate, which can be obtained by the following Equation (1).

(1) $$T_{V}^{PR}=T_{PA}^{PR}*T_{V}^{PA}={\left(T_{PR}^{O}\right)}^{-1}*T_{PA}^{O}*T_{V}^{PA}$$

In order to show the operation interaction in the projection, the surgical tool needs also to be transformed into the projector coordinate in Equation (2). At the same time, the coordinate of the surgical tool is unified in the patient space, thus, information such as the deviation of the surgical tool from the planned surgical path can be provided, which can be determined in Equation (3).

(2) $$T_{S}^{PR}={\left(T_{PR}^{O}\right)}^{-1}*T_{S}^{O}$$

(3) $$T_{S}^{PA}={\left(T_{PA}^{O}\right)}^{-1}*T_{S}^{O}$$

The calibrations between projector’s lens and marker, surgical tool’s tip and marker are described in the next step.

### 3.3. Projector Calibration

**Figure 4:**
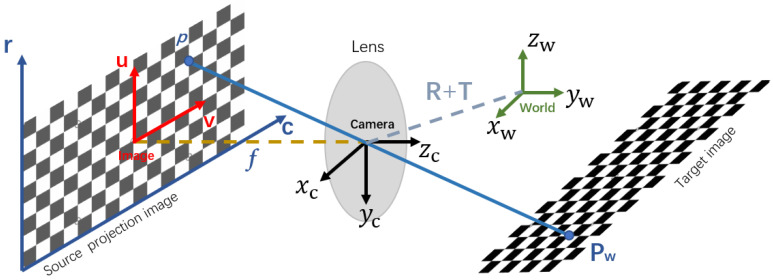
The inverse camera pinhole model of projector for calibration.

The projector can be assumed as an inverse camera as a pinhole model according to Hartley et al. [30]. Contrary to the camera capturing and generating an image from the light entering the small hole, the projector emits light out from the small hole according to the source image, and casts the image into the environment. The relationship between a projection point on a spatial surface and its corresponding pixel on the source image is referred to as perspective projection, as described in Figure 4.

The mapping from point in 3D space to 2D image pixel can be modeled using Zhang’s model [31] as follows,

(4) sp=KTP

(5) $$K=\left[\begin{array}{ccc} f_{x} & \alpha & c_{x}\\ 0 & f_{y} & c_{y}\\ 0 & 0 & 1 \end{array}\right]$$

(6) $$T=\left[\begin{array}{cc} R & t \end{array}\right]$$

where $p={\left[\begin{array}{ccc} u & v & 1 \end{array}\right]}^{T}$ is the position of the pixel on the source image plane and $P={\left[\begin{array}{cccc} X & Y & Z & 1 \end{array}\right]}^{T}$ is the position of pixel’s corresponding projection point on the spatial surface. The extrinsic matrix *T* includes the rotation of a 3-by-3 matrix *R* and the translation of a 3-by-1 vector *t*, which together represent the transformation from the real-world space to the projector’s lens coordinate system. The intrinsic matrix *K* contains the focal length along the X and Y axis specified as $\left[\begin{array}{cc} f_{x} & f_{y} \end{array}\right]$, the camera’s principle point’s position along the X and Y axis specified as $\left[\begin{array}{cc} c_{x} & c_{y} \end{array}\right]$, and skew of the image axes α, and the scale factor *s*, which is related to the depth from the projection surface to the projector.

#### 3.3.1. Intrinsic Matrix Calculation

This pinhole projector model allows one to apply known camera calibration methods to calibrate the projector. Here, Zhang’s method is applied [31]. The corresponding point-pixel pairs at checkerboard corners are required to solve the model. However, the projector can only cast the source image but cannot acquire the projection image, and thus, a camera (Hikvision, China) is used to help to find the point-pixel pairs. Due to the difference between the viewpoints’ position and resolution of the projector and the camera, the image captured by the camera is not the same as the image projected by the projector.

**Figure 5:**
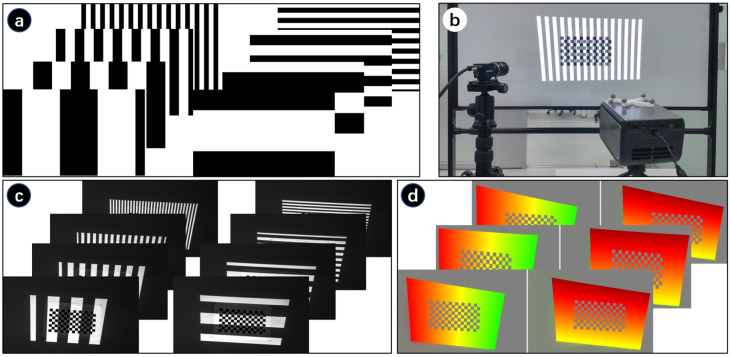
Gary code projection. (**a**) Source gray code patterns; (**b**) Project gray code and capture; (**c**) Captured projection; (**d**) Decoded captured images.

Here, gray code is used to find the correspondence between pixels captured by camera and pixels casted by the projector. Source gray code patterns are generated according to the encoding principle of gray code, as shown in Figure 5a. The number of the patterns is related to the resolution of the projector, which equals $2(log_{2}width+log_{2}height)$. The sequence of these patterns codes unique locations.

A printed checkerboard is pasted on a whiteboard and then the source gray code patterns are sequentially casted to cover the printed checkerboard, as shown in Figure 5b. Then, we use the camera to take pictures of the projected gray code patterns. The captured images are shown in Figure 5c. Change the whiteboard’s position and repeat projection and capturing. Then, we can obtain sequences of captured gray code projection from different perspectives. Each pixel of the projector can be identified with a unique black/white sequence. The mapping between projector’s pixels and camera’s pixels can be established by decoding the captured gray code images, as shown in Figure 5d. Each projection pixel’s 2D location can be recovered by decoding the patterns. Usually, the resolution of the camera is larger than that of the projector in order to take better quality image for analysis, so that the mapping is not bijective. According to Moreno [27], we applied local homography transformation to achieve better mapping. By detecting the checkerboard corners from the captured gray code image, we can get the corners’ pixel coordinate from the camera’s perspective. With camera-projector mapping, we can get the corners’ pixel coordinate from the projector’s perspective. Finally, the point-pixel pairs are obtained and the intrinsic parameters of projector are calculated by applying Zhang’s method.

#### 3.3.2. Extrinsic Matrix Calculation

**Figure 6:**
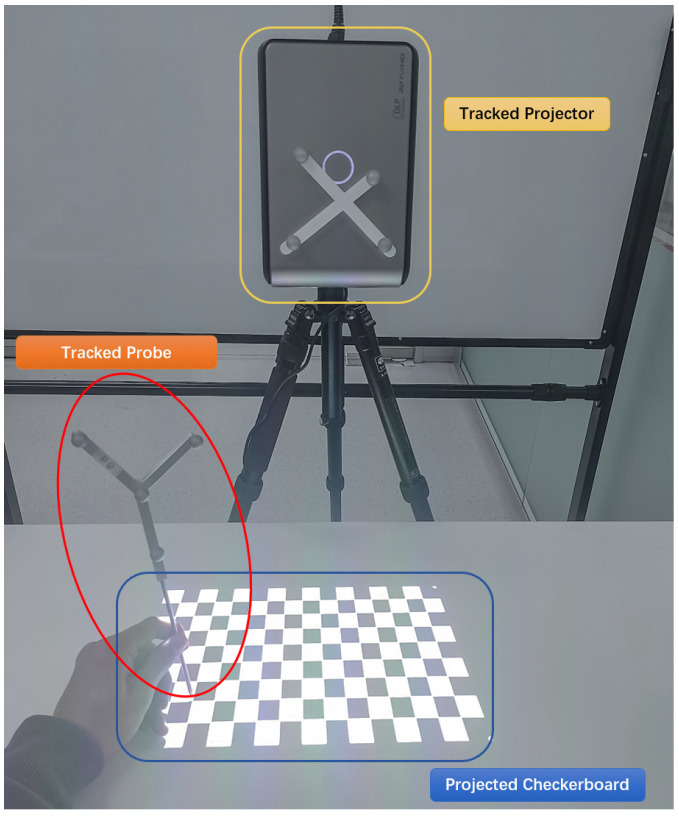
Obtain the spatial position of the projection point.

With the intrinsic parameters of the projector known, the next step is to find the extrinsic parameters, which indicates the transformation between the projector’s lens and the OTS. We render a checkerboard on computer screen which is then projected onto a flat surface using the projector. A marker was fixed to the projector to be set as the world coordinate reference, which makes sure that all coordinates in the inverse camera pinhole model remain unchanged. The 3D positions of the projected checkerboard corners are obtained by digitization using a tracked probe, which are transformed to the world coordinate space, as shown in Figure 6. Pairs of corresponding point-pixels were generated from projections, which cover the potential workspace of the projector.

Considering that there is noise in Equation (4), the error between the projection position of the 3D point and the observation pixel’s position can be described as a function f(T), as shown in Equation (7):

(7) $$f\left(T\right)=u_{i}-\frac{1}{s_{i}}KTP_{i}$$

By minimizing the error f(T), we can construct a least squares problem, and find the best extrinsic parameter matrix $T^{*}$, as follows:

(8) $$T^{*}=\underset{T}{\arg\min}\frac{1}{2}\sum_{i=1}^{n}{∥f\left(T\right)∥}_{2}^{2}$$

By performing a first-order Taylor expansion of f(T), we can obtain Equation (9).

(9) $$f\left(T+\Delta T\right)\approx f\left(T\right)+J{\left(T\right)}^{T}\Delta T$$

where the $J{\left(T\right)}^{T}$ is the derivative of f(T) with respect to *T*, if *T* is in superscript, it means the symbol of matrix transposition. The target is to find a ΔT to minimize the ${∥f\left(T+\Delta T\right)∥}^{2}$, as shown in Equation (10).

(10) $${∥f\left(T\right)+J\left(T\right)}^{T}{\Delta T∥}^{2}={∥f\left(T\right)∥}_{2}^{2}+2f\left(T\right)J{\left(T\right)}^{T}\Delta T+\Delta T^{T}J\left(T\right)J{\left(T\right)}^{T}\Delta T$$

Find the derivative of ${∥f\left(T\right)+J\left(T\right)}^{T}{\Delta T∥}^{2}$ with respect to ΔT, and set the derivative to zero. We can obtain the following Equation (11).

(11) $$J\left(T\right)f\left(T\right)+J\left(T\right)J{\left(T\right)}^{T}\Delta T=0$$

Transform the 3D point from world coordinate space *P* to projector’s lens space $P^{\prime }$, as follows:

(12) $$P^{\prime }=TP={\left[X^{\prime },Y^{\prime },Z^{\prime }\right]}^{T}$$

Substitute Equation (12) into Equation (4), then we can get:

(13) $$su=KP^{\prime }$$

(14) $$s\left[\begin{array}{c} u\\ v\\ 1 \end{array}\right]=\left[\begin{array}{ccc} f_{x} & 0 & c_{x}\\ 0 & f_{y} & c_{y}\\ 0 & 0 & 1 \end{array}\right]*\left[\begin{array}{c} X^{\prime }\\ Y^{\prime }\\ Z^{\prime } \end{array}\right]$$

From Equation (14), we can get:

(15) $$u=f_{x}\frac{X^{\prime }}{Z^{\prime }}+c_{x}$$

(16) $$v=f_{y}\frac{Y^{\prime }}{Z^{\prime }}+c_{y}$$

According to the chain rule of derivatives, we can get:

(17) $$J\left(T\right)=\frac{\partial f(T)}{\partial P^{\prime }}*\frac{\partial P^{\prime }}{\partial T}$$

(18) $$\frac{\partial f(T)}{\partial P^{\prime }}=-\left[\begin{array}{ccc} \frac{f_{x}}{Z^{\prime }} & 0 & -\frac{f_{x}X^{\prime }}{Z^{\prime 2}}\\ 0 & \frac{f_{y}}{Z^{\prime }} & -\frac{f_{y}Y^{\prime }}{Z^{\prime 2}} \end{array}\right]$$

We set the Lie algebra corresponding to ΔT as δξ. According to the disturbance model, we multiply *T* left by a disturbance ΔT, then we can get:

(19) $$\frac{\partial (TP)}{\partial \delta \xi }=\lim_{\delta \xi \to 0}\frac{exp\left(\delta \xi^{\wedge }\right)exp\left(\xi^{\wedge }\right)P-exp\left(\xi^{\wedge }\right)P}{\delta \xi }=\left[\begin{array}{cc} I & -{\left(RP+t\right)}^{\wedge }\\ 0^{T} & 0^{T} \end{array}\right]$$

where ${\left(RP+t\right)}^{\wedge }$ indicates the antisymmetric matrix of (Rp+t). So, the *J* is as follows:

(20) $$J\left(T\right)=-\left[\begin{array}{cccccc} \frac{f_{x}}{Z^{\prime }} & 0 & -\frac{f_{x}X^{\prime }}{Z^{\prime 2}} & -\frac{f_{x}X^{\prime }Y^{\prime }}{Z^{\prime 2}} & f_{x}+\frac{f_{x}X^{\prime 2}}{Z^{\prime 2}} & -\frac{f_{x}Y^{\prime }}{Z^{\prime }}\\ 0 & \frac{f_{y}}{Z^{\prime }} & -\frac{f_{y}Y^{\prime }}{Z^{\prime 2}} & -f_{y}-\frac{f_{y}Y^{\prime 2}}{Z^{\prime 2}} & \frac{f_{y}X^{\prime }Y^{\prime }}{Z^{\prime 2}} & \frac{f_{y}X^{\prime }}{Z^{\prime }} \end{array}\right]$$

To solve the ΔT in Equation (11), we give an initial value $T^{\prime }$, calculate the current *J* and error *f*, and then find ΔT. If δξ corresponding to ΔT is small enough, the iteration stops. Otherwise, the *T* is continuously updated by multiply ΔT left and iterated.

Let’s make the final extrinsic matrix

(21) $$T^{*}=\left[\begin{array}{cccc} X^{*} & Y^{*} & Z^{*} & P^{*}\\ 0 & 0 & 0 & 1 \end{array}\right]$$

where the $X^{*},Y^{*},Z^{*},P^{*}$ are 3 by 1 vectors. The $Y^{*},Z^{*},P^{*}$ indicate the view down direction, the center line direction and the optical center of the projector. The $c_{x},c_{y}$ indicates the window center corresponding to the optical center. All the parameters are used to set the render environment.

### 3.4. Surgical Tool Calibration

To involve surgical tool in the AR scene, we need to calibrate the tool. For puncture needle, it can be represented by a tip and axis. A fixture was designed to rigidly connect the surgical tool with markers, which can be tracked as the tool’s reference.

**Figure 7:**
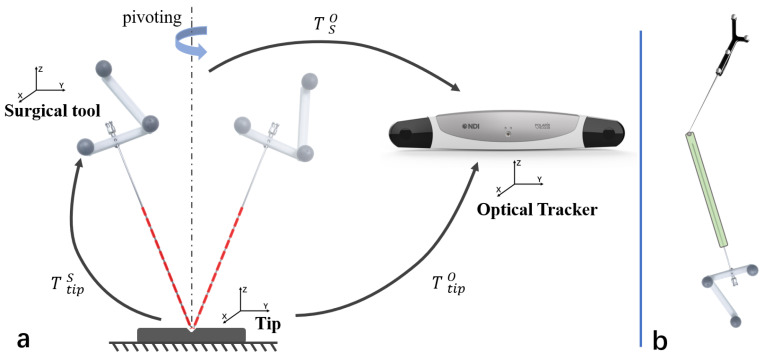
Calibrate the puncture needle. (**a**) Pivot the needle to find the offset of the tip; (**b**) Find the axis’ direction using the probe.

The offset of the tool’s tip could be computed by pivoting the tool, as described in Figure 7a. The relationship of transformations can be denoted as below.

(22) $$T_{S}^{O}*T_{Tip}^{S}=T_{Tip}^{O}$$

The transformations could be decomposed into blocks as bellow:

(23) $$\left[\begin{array}{cc} R_{S}^{O} & t_{S}^{O}\\ 0 & 1 \end{array}\right]*\left[\begin{array}{cc} R_{Tip}^{S} & t_{Tip}^{S}\\ 0 & 1 \end{array}\right]=\left[\begin{array}{cc} R_{Tip}^{O} & t_{Tip}^{O}\\ 0 & 1 \end{array}\right]$$

where the *R* means the rotation and the *t* means the translation. Using the Equation (23), we could have:

(24) $$R_{S}^{O}*t_{Tip}^{S}+t_{S}^{O}=t_{Tip}^{O}$$

which could be reorganized as follows:

(25) $$\left[\begin{array}{cc} I & -R_{S}^{O} \end{array}\right]*\left[\begin{array}{c} t_{Tip}^{O}\\ t_{Tip}^{S} \end{array}\right]=t_{S}^{O}$$

with pivoting the surgical tool while the optical tracker tracking, a large set of pairs data $\left({R_{S}^{O}}_{i}|{t_{S}^{O}}_{i}\right)$ could be given. Then the equation will be:

(26) $$\left[\begin{array}{cc} I & -{R_{S}^{O}}_{1}\\ \vdots & \vdots \\ I & -{R_{S}^{O}}_{n} \end{array}\right]*\left[\begin{array}{c} t_{Tip}^{O}\\ t_{Tip}^{S} \end{array}\right]=\left[\begin{array}{c} {t_{S}^{O}}_{1}\\ \vdots \\ {t_{S}}_{n}^{S} \end{array}\right]$$

We can find a least square fit solution of ${\left[\begin{array}{cc} t_{Tip}^{O} & t_{Tip}^{S} \end{array}\right]}^{T}$.

The tip’s offset from the tool’s coordinate system $t_{Tip}^{S}$ was obtained. To calibrate the axis of the tool, a cylindrical sleeve was designed to be fixed on the needle. As shown in Figure 7b, using the tracked probe to point out a hemispherical groove on the sleeve, which is coincided with the tool’s axis, then the axis’s direction can also be computed. With the tip and axis known, the tool can be modeled.

### 3.5. Patient Registration

**Figure 8:**
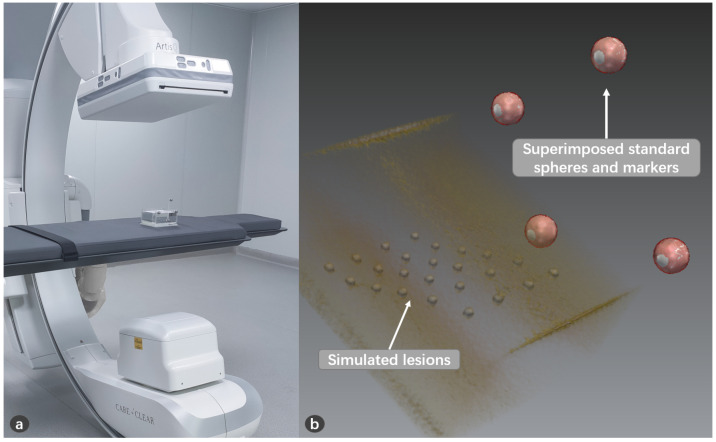
The patient registration. (**a**) CT scan the patient together with sphere markers; (**b**) CT image registered with sphere markers.

To align the surgical plan to projector’s reference, the patient is rigidly registered into the system. We choose paired-points matching, which is a common method for the rigid registration [32]. To find the corresponding pair-points, patient is scanned together with passive sphere markers. CT images containing patient and sphere markers are acquired together. Surface matching is applied to match standard sphere model with sphere markers to locate the position of sphere markers in CT image space, as shown in Figure 8. The sphere markers’ coordinates are already measured by infrared in OTS space. So, we can establish the connection between image space and OTS space, which is denoted by $T_{V}^{PA}$. Finally, the virtual images can be displayed in the coordinate space of the projector.

### 3.6. Interactive Display Design

VTK(The Visualization Toolkit, Kitware, Inc., USA) is used to build the rendering environment. The virtual vtk camera is set with the calibrated projector’ parameter, including the principle point, the focus direction, the view up direction and the window center, which means the position of the lens center on the screen. The patient’s polygon model could be reconstructed from the segmenteation of preoperative CT image. As soon as any patient is registered to the projector’s reference, the polygon model can be displayed by the projector.

**Figure 9:**
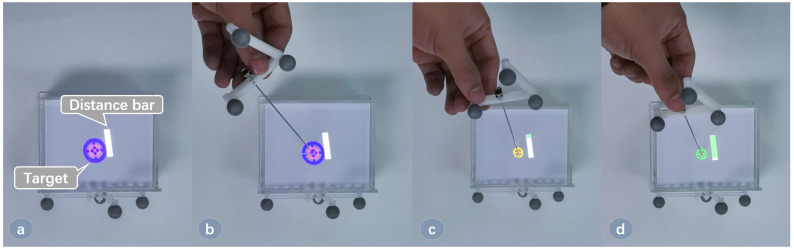
The augmented view for puncture. (**a**) Project the circles on the surface; (**b**) Align the needle’s tip to the center of the circle; (**c**) Adjust the direction of the needle to the circle turning yellow; (**d**) Start the puncture, and when the target turns green, it means that the needle has reached the lesion.

We mainly studied the application scenarios of the projection in puncture surgery. During the puncture, two circles are projected onto the surface, one representing the target lesion to be punctured, and the other representing the axis of the surgical needle. A bar aside representing the distance between the surgical needle’s tip and the target is also projected, as shown in Figure 9. The surgeon aligns the needle’s tip to the center of a projected magenta circle, and there is another blue circle indicating the direction of the needle. When the needle is rotating to the right direction, the blue circle will shrink. When the axis of the needle is aligned with the target, the circles will turn yellow. Then, the surgeon inserts the needle in the direction. As the needle gets closer to the target, the green distance bar will get longer. Finally, a full green bar indicates that the needle has reached the lesion, and the circles turn green to remind the surgeon.

## 4. Validation Experiments

Experiments were designed to validate the performance of the proposed system.

### 4.1. Projection Accuracy Validation

**Figure 10:**
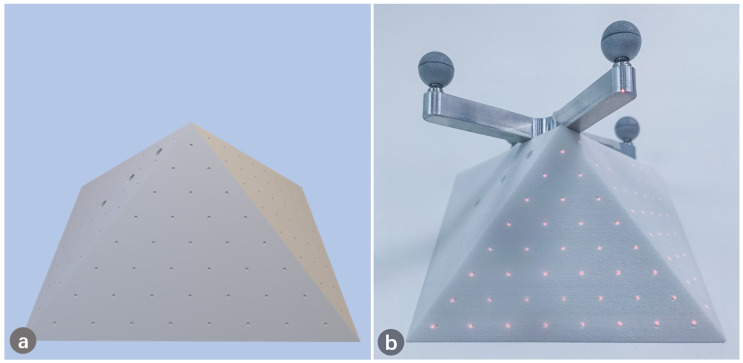
Points projected onto the 3D-printed pyramid for projection accuracy validation. (**a**) The designed pyramid with points; (**b**) Points were projected to overlap the pyramid.

To evaluate the accuracy of the projection, a phantom model with pyramid shape was designed and 3D printed. Points were pre-designed evenly on the surface of the pyramid, as shown in Figure 10a. The location of each designed point on the pyramid was known in advance. We first projected the generated points to the phantom target, as shown in Figure 10b. We then used a tracked probe to obtain the position of the projected points and computed the distance between the projected and designed points, which indicates the accuracy of overlapping. We further converted the coordinates of projected 3D points into pixels, and compared them with the designed pixel coordinates.

### 4.2. Puncture Accuracy Validation

**Figure 11:**
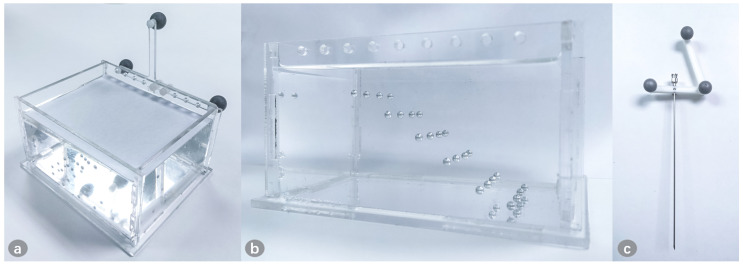
The model for puncture. (**a**) The model is fixed with the marker, and is covered to ensure that the surgeon cannot see the internal lesion information from above; (**b**) The lesions are placed at different depths, and the side of the model is transparent to check whether the needle successfully reaches the lesion; (**c**) The puncture needle is fixed with the 3D-printed reference.

A model was made for puncture experiment using transparent silicone, in which aluminum balls of 3mm diameter were placed at different depths as simulated lesions, as shown in Figure 11. The model was covered to hide the lesions. The lesions (aluminum balls) were segmented from CT image. A volunteer used a calibrated needle to puncture the lesion. The volunteer can only perform the puncture from the covered side to avoid seeing the lesions. After the operation, we can check whether the needle reaches the lesion through the transparent material on the side. The success rate of the puncture is recorded.

### 4.3. Visual Effect Evaluation

**Figure 12:**
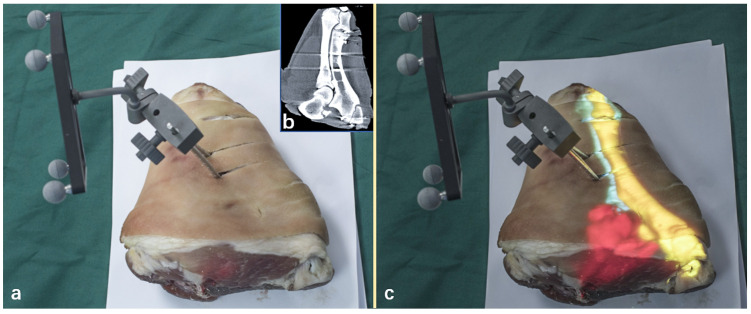
Projection on the in vitro pig leg. (**a**) The original pig leg without projection; (**b**) The CT image indicating the internal bone; (**c**) Project the internal anatomy onto the pig leg.

An in vitro pig leg was set as the projection target to simulate the performance on real skin. The image data were obtained through CT scanning. After segmentation and reconstruction, the underlying bony structures were projected onto the skin of the pig leg to qualitatively demonstrate the projection, as shown in Figure 12.

Surgeons were invited to rate the performance of system with filling in a designed questionnaire, which contains 10 questions for the evaluation of the system. Each question scores in tenths. The results of the questionnaire were statistically analyzed to evaluate the visual performance of the system on real skin.

## 5. Results

**Figure 13:**
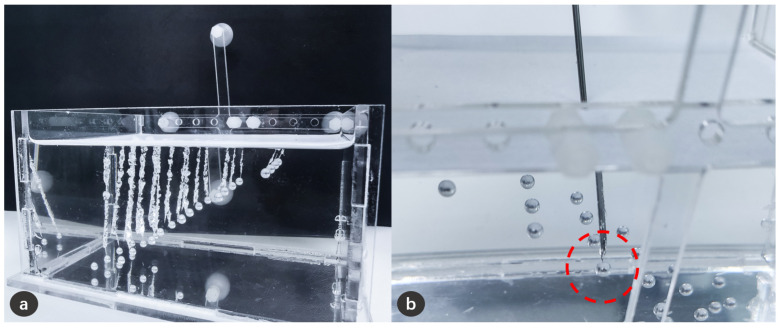
Schematic diagram of puncture experiment results. (**a**) The trajectories formed on the silicone after needle puncture, and these trajectories have successfully reached the lesion; (**b**) It can be clearly confirmed from the side that the needle has reached the lesion.

**Figure 14:**
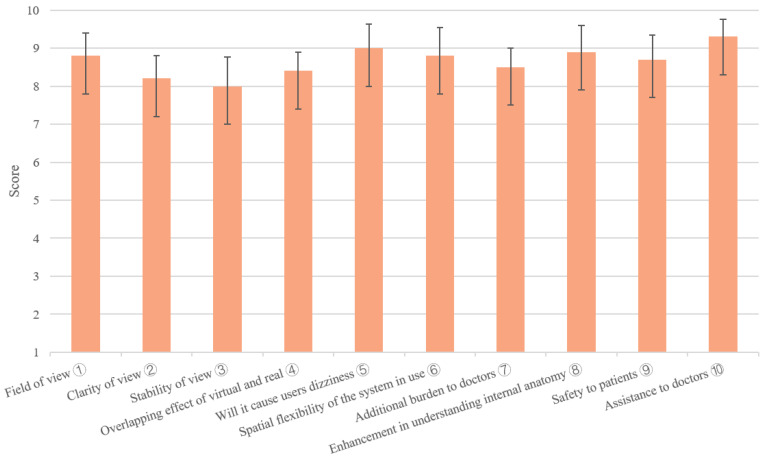
Results of the qualitative analysis (➄ higher score means less dizziness; ➆ higher score means less burden).

In total, 5 experiments of projection accuracy measurement were carried out, and 200 punctures were conducted, and 10 questionnaires from doctors were collected. We collected 120 3D points to convert into pixel coordinates, and found that the average back projection error is 3.37 pixels in *x* direction and 1.51 pixels in *y* direction. For the projection on the pyramid model, an average projection error of 1.03 ± 0.43 mm was found, as shown in Table 1. For the puncture experiment, the success rate reached 99%, as shown in Figure 13. For the visual effect evaluation study, the results of the questionnaires are shown in Figure 14.

## 6. Discussion

In this study, we proposed a projector-based AR navigation system and investigated the feasibility of the system’s application in surgery. Our AR system helps to link preoperative planning with the intraoperative surgical scenario.

In the proposed system, the navigation data could be accurately projected onto the surgical scenario. During the surgery, the navigation system could guide the surgeon to avoid areas of risk, find a specific anatomical target, and offer intraoperative orientation in the absence of anatomical landmarks, which makes the operation more effective, safer and less invasive. Our projector-based AR system could be treated as an improved alternative to monitor-based navigation, which could help the surgeon to avoid diverting attention around the monitor and the patient, which may cause the loss of precision and increase the risk caused by distraction [33]. Furthermore, comparing to other alternative technologies (head-mounted displays, see-through screens), the described system could provide the same AR view of navigation data to all surgical teams, including surgeons and assistants, and the surgeons do not need to put burdens on themselves, which greatly releases the pressure on surgeons [34].

By projecting the gray code and using the camera to capture the projected image, we can analyze and decode the projected point-pixel pairs from the perspective of the projector. Thus, we can apply the method of camera calibration to the projector. By minimizing the back-projection error, we established a least squares problem, and used the Newton–Gauss method and Lie group and Lie algebra theory to optimize the extrinsic parameter matrix of the projector iteratively. Due to manufacturing errors, the center axis of the lens is not strictly aligned with the center of the image sensor, and the deviation between the optical axis and the image center can be obtained through intrinsic parameters. When setting up the rendering environment, we took this factor into consideration and compensated for this deviation by setting the window center.

The image and navigation information is projected from the perspective of the projector, which is different from the perspective of users. Parallax exists due to the different viewpoints between the user and the projector. Especially the deeper the target from the projection surface, the greater the deviation, which has also been reported by other researchers [21]. In order to eliminate the influence of this parallax, we have also tracked the surgical tools and involed them in the projection. We mainly carried out feasibility studies of the system in puncture surgery, which requires a needle to be inserted into the lesion. We have designed graphics guidance interface in the application, and the experimental results prove that it works well. Furthermore, during the puncture experiment, we tried punctures to lesions of different depths, and the results show that even deep lesions can be reached with guidance, which shows that connecting the surgical tool to the projected guidance information can eliminate the effect of depth.

In the operating room, there may be many necessary devices surrounding the patient, such as ventilator and anesthesia machine, so that there may be little space spare and the devices may change their position intraoperatively, so it is inevitable to adjust the position of components in the navigation system. In our study, once the calibration and registration are finished, all the components in the system can be relocated to meet the need of the surgery, with the projected guidance information always following the patient.

The benefit of overlaying the navigation data onto patients is apparent, however, the environment has a great influence on the effect of the system [15]. Bright environment will make the projected image difficult to recognize. The material of the projected surface also has a great influence on the display effect of the projection, for example, due to the absorption of light, the performance on surface of light colors would be better than that of dark colors. The projector we use has 1100ANSI lumens, of which the light intensity is sufficient to provide distinguishable image guidance in the operating room. By projecting on real skin and inviting doctors to score the system, we evaluated the performance of the system. The results show that the system has sufficient clarity and good performance when projecting on real skin, and can help doctors understand the internal anatomy. In the puncture application, the deformation of the soft tissue and the surgical needle’s bending could lead to errors, which is also a challenge that requires investigation.

In the future, the clinical application with the proposed system will be performed.

## 7. Conclusions

In this paper, we developed a projector-based AR navigation system for computer-assisted surgery which provides AR guidance information during operation. Our experiments demonstrate the efficacy of the proposed system.

## Figures and Tables

**Table 1:** Projection errors in position of the experiment results.

Projection	1	2	3	4	5	Total
Position error (mm)	1.16 ± 0.55	0.97 ± 0.31	1.08 ± 0.43	1.04 ± 0.41	0.92 ± 0.39	1.03 ± 0.43

## Data Availability

Not applicable.
